# Variations in the Gene Expression Profile in Atherosclerotic Patients with Non-Fatal ACS: A Preliminary Study

**DOI:** 10.3390/ijms23095017

**Published:** 2022-04-30

**Authors:** Angela Dziedzic, Rafal Szelenberger, Michal Kacprzak, Piotr Czarny, Ewelina Synowiec, Joanna Saluk-Bijak, Tomasz Sliwinski, Marzenna Zielinska, Michal Bijak

**Affiliations:** 1 Department of General Biochemistry, Faculty of Biology and Environmental Protection, University of Lodz, Pomorska 141/143, 90-236 Lodz, Poland; angela.dziedzic@edu.uni.lodz.pl (A.D.); rafal.szelenberger@edu.uni.lodz.pl (R.S.); 2 Biohazard Prevention Centre, Faculty of Biology and Environmental Protection, University of Lodz, Pomorska 141/143, 90-236 Lodz, Poland; michal.bijak@biol.uni.lodz.pl; 3 Intensive Cardiac Therapy Clinic, Medical University of Lodz, Pomorska 251, 91-213 Lodz, Poland; michal.kacprzak@umed.lodz.pl (M.K.); marzenna.zielinska@umed.lodz.pl (M.Z.); 4 Department of Medical Biochemistry, Medical University of Lodz, Mazowiecka 6/8, 92-215 Lodz, Poland; piotr.czarny@umed.lodz.pl; 5 Laboratory of Medical Genetics, Faculty of Biology and Environmental Protection, University of Lodz, Pomorska 141/143, 90-236 Lodz, Poland; ewelina.synowiec@biol.uni.lodz.pl (E.S.); tomasz.sliwinski@biol.uni.lodz.pl (T.S.)

**Keywords:** platelet–leukocyte aggregates, platelet–monocyte aggregates, blood platelets, atherosclerosis, acute coronary syndrome, cytokines, inflammation

## Abstract

The pathophysiology of atherosclerosis and acute coronary syndrome (ACS) is related to interactions between immune cells, endothelium, and blood platelets. An increasing number of reports confirm the link between excessive immune activation and cellular cross-talk with ACS incidence. Our genetic and proteomic analysis was performed on strictly selected atherosclerotic patients with non-fatal ACS without typical risk factors and healthy donors. Results showed changes in the gene expression levels of the various inflammatory factors derived from the peripheral blood cells that drive the over-activation of the immune system. The enhanced activation of the immune system may lead to the overexpression of the pro-inflammatory mediators, which causes self-perpetuating machinery of processes associated with thrombosis. In our preliminary study, we confirmed an altered expression of genes associated with the inflammation and overall interaction of the vascular microenvironment. Furthermore, 5 of 92 analyzed genes, *CCL2*, *CCR2*, *CSF2*, *GZMB*, and *ICOS*, were expressed only in patients with ACS. In conclusion, the augmented expression of the pro-inflammatory genes from the peripheral blood cells may be a crucial genetic factor leading to the occurrence of acute inflammation and thus be significant in ACS pathogenesis.

## 1. Introduction

Cardiovascular diseases, including acute coronary syndrome (ACS), are one of the most life-threatening disorders responsible for cause-specific high mortality and morbidity in the world. According to the World Health Organization (WHO), approximately 17.9 million people worldwide died from cardiovascular diseases in 2019, making it the leading cause of death globally [1]. ACS is a term that encompasses patients with a broad spectrum of symptoms caused by blockage of blood flow in coronary arteries and includes ST-elevation myocardial infarction (STEMI), non-ST-elevation myocardial infarction (NSTEMI), and unstable angina (UA). Although thrombosis is the direct cause of acute coronary events, atherosclerosis is the persistent disease underlying most myocardial infarctions and strokes. Increased platelet activation and aggregation in ruptured atherosclerotic plaques are associated with thrombotic coronary occlusion, being a critical mechanism leading to acute myocardial infarction [2]. In addition, partial or complete occlusion of the blood vessel lumen interrupts a blood supply in the coronary circulation resulting in myocardial ischemia. Atherosclerosis and its complications are well known for their complexity and multifactorial character. Moreover, current literature indicates that atherosclerosis and ACS are more often observed in patients without typical risk factors [3,4]. In recent years, many studies provided consistent evidence that blood platelets, besides driving thrombus formation at the site of atherosclerotic plaque rupture, also play a pivotal role in the inflammatory response. Furthermore, increasing evidence shows that interactions between platelets and immune cells play an essential role in atherogenesis progression and the pathophysiology of ACS [5].

The chronic activation and increased hemostatic activity of platelets in ACS and atherosclerosis are proven [6,7], while their role in inflammation appears to be relatively neglected and still needs to be clarified. The functions and activity of lymphocytes and platelets are mutually regulated by direct cell–cell contact or via soluble mediators. Blood platelets possess a large variety of surface receptors, which interact with immune cells. They are also a rich source of secretory molecules, which are mediators of inflammation and immunity [8]. Pathologically activated blood platelets demonstrate overexpression of biologically active molecules stored in intracellular granules of resting platelets, including membrane receptors (e.g., GPIIb-IIIa, GPIb-IX-V, GPVI, and P-selectin) [9,10,11,12,13], adhesion proteins (e.g., fibrinogen, von Willebrand factor (vWF), and thrombospondin) [14,15,16], serotonin (5-hydroxytryptamine, 5-HT) [17], and growth factors (e.g., epidermal growth factor (EGF) [18], transforming growth factor β (TGF-β)) [19,20]. The pro-inflammatory factors headed by chemokines, like CXCL4 (platelet factor 4, PF-4) [21], recruit leukocytes to inflammatory sites by inducing activation of integrins present on the monocyte surface and promoting infiltration of the macrophage to the vascular wall [22]. 

Inflammatory processes accompany all stages of atherogenesis, from the early phases of lesion formation to plaque disruption. Inflammation of the vascular wall is characterized by cell cross-talk, which triggers the autocrine and paracrine activation processes that lead to leukocyte movement into the disrupted vessel wall. A vast amount of platelet- and leukocyte-derived secretory molecules mediate the cells′ interactions in the early stages of atherosclerosis. The reperfusion area is the most particularly exposed to increased accumulation of immune cells, which play a significant role in myocardial injury at the damaged tissue site with re-infiltration of oxygen. The permanent stimulation of immune cells is vital for developing atherosclerosis and its possible complications [23]. Several reports confirmed that plasma levels of several markers of inflammation had been associated with atherosclerosis progression and increased cardiovascular risk in various clinical settings [24,25]. 

Chemokines and pro-inflammatory cytokines have a significant influence on all prothrombotic and pro-atherogenic pathways [26]. The importance of inflammation in atherosclerosis was confirmed in the CANTOS clinical trial, which showed that anti-inflammatory therapy focused on targeting interleukin 1β (IL-1β) significantly decreased the rate of cardiovascular events [27]. The dependence between pro-inflammatory cytokines and the over-activation of platelets in the ACS pathogenesis was also observed [28,29], thus indicating the similarity of background in atherosclerosis complications. This preliminary study aimed to investigate the blood platelets and leukocytes interactions and cognize the contribution of pro-inflammatory factors well known in atherosclerosis patients with non-fatal ACS complications without typical risk factors.

## 2. Results

### 2.1. Formation of Platelet–Leukocyte Aggregates (PLAs) and Expression of P-Selectin Measured by Flow Cytometry 

**Figure 1:**
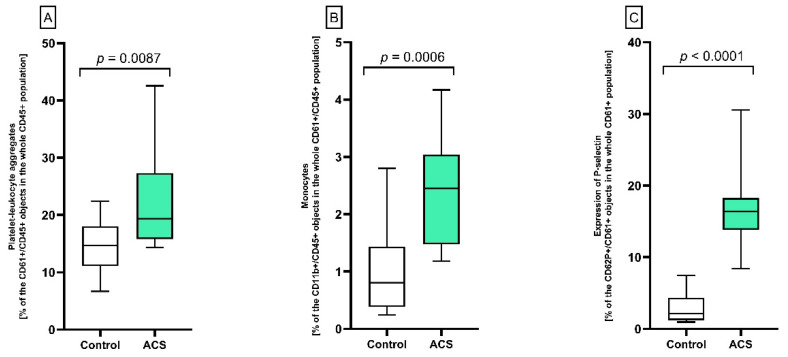
Analysis of PLA formation, monocyte participation, and platelet activation using double-label flow cytometry. The cells were distinguished based on the expression of CD61/FITC (platelets) or CD45/PE (leukocytes). For each sample, 15,000 CD61+ or CD45+ objects were acquired. (**A**) The formation of PLAs is shown as the percentage of CD61+/CD45+ objects in the whole CD45+ population. (**B**) Contribution of monocytes in whole CD61+/CD45+ population. Results are shown as the percentage of monocytes (CD11b+/CD45+ objects in the whole population of cells forming PLAs). (**C**) To assess CD62P (P-selectin) expression, samples were labeled with fluorescently conjugated monoclonal antibody CD62/PE, and the results are shown as the percentage of platelets expressing CD62P antigen (CD61+/CD62P+ objects in the whole CD61+ population). The results are represented on the box plots with the midpoint as median (the frame—the interquartile range quartiles: Q1–Q3; whiskers—the minimum and maximum value, respectively), *n* = 12. Statistical analysis was performed using the Mann–Whitney U test.

To determine the level of interactions between platelets and leukocytes in whole blood samples, the flow cytometry method utilizing the antibodies specific for platelets (anti-CD61+) and leukocytes (anti-CD45+) was used. The obtained results indicated a statistically significant elevated number of the formed PLAs in patients with ACS compared to healthy controls (*p* = 0.0087; 19.34 vs. 14.67%) (Figure 1A). Moreover, it was observed that among the leukocytes involved in the formation of PLAs complexes, the monocyte population increased significantly (*p* = 0.0006) in ACS compared to the control group (2.45 vs. 0.81%, respectively) (Figure 1B). In cytometry measurements, the level of surface expression of P-selectin on blood platelets was statistically significantly higher (*p* < 0.0001) in ACS patients compared to the control group (16.37 vs. 2.12%, respectively) (Figure 1C).

### 2.2. The Concentration of PF-4 and Soluble (s)P-Selectin in Human Plasma

**Figure 2:**
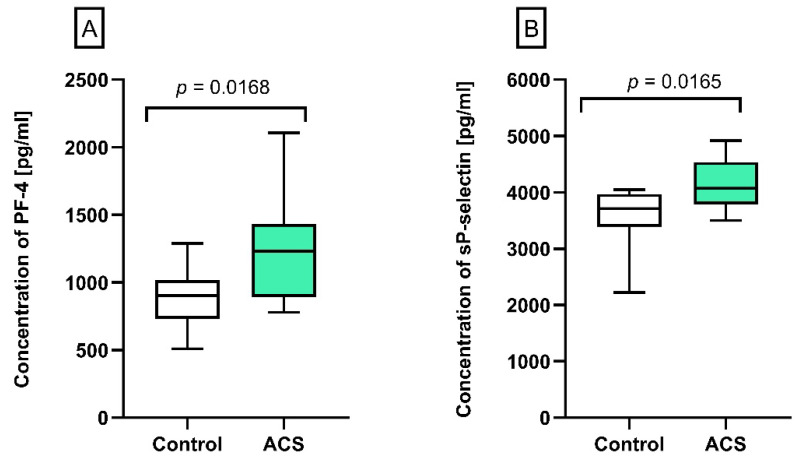
Analysis of blood platelet activation markers in plasma samples. (**A**) The concentration of PF-4 [pg/mL] and (**B**) sP-selectin [pg/mL] were measured by the ELISA test in plasma from patients with ACS (*n* = 12) and healthy subjects (*n* = 12). The results were presented as the median with an interquartile range (Q1–Q3; whiskers—the minimum and maximum value, respectively). Statistical analysis was performed using the Mann–Whitney U test.

The plasma concentration of PF-4 and sP-selectin was determined using the ELISA method. Obtained results showed a significantly augmented concentration of PF-4 (*p* = 0.0168, 1231.5 pg/mL vs. 901.5 pg/mL, Figure 2A) and sP-selectin (*p* = 0.0165, 4070 pg/mL vs. 3710 pg/mL, Figure 2B) in plasma from ACS patients compared to the control group.

### 2.3. Pro-Inflammatory Cytokines Measurement in Human Plasma

The plasma concentrations of pro-inflammatory cytokines were determined using the ELISA method. For this purpose, six of the most significant cytokines involved in cellular immune responses [30,31]—IL-1β, IL-2, interferon (IFN-)α, IFN-γ, tumor necrosis factor (TNF-)α, and TGF-β—were designated. A significantly higher level of IL-1β, IL-2, INF-γ, and TNF-α in ACS patients was observed (Table 1). Based on the obtained results, the greatest fold-change between studied groups was shown for IL-1β (over 2-fold increase relative to controls, 21.7 pg/mL vs. 45.5 pg/mL, *p* = 0.0003, Table 1) and TNF-α (2-fold increase relative to controls, 356.33 pg/mL vs. 752.33 pg/mL, *p* < 0.0001, Table 1).

### 2.4. The Analysis of IL1B and TNFA Gene Expression by Real-Time PCR

**Figure 3:**
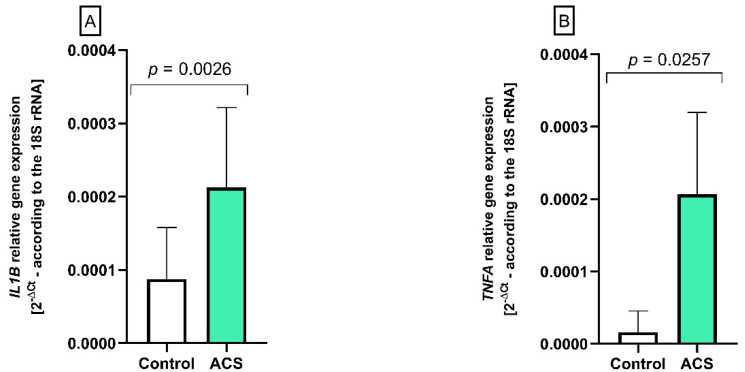
The altered expression of (**A**) *IL1B* and (**B**) *TNFA* genes in the peripheral blood cells from patients with ACS compared to healthy subjects. The results from gene expression are presented as a mean of 2^−ΔCt^ (according to the gene encoding 18S rRNA) ± SD, *n* = 12. Statistical analysis was performed using Student’s *t*-test.

Based on the fold-change values from protein concentration of inflammatory cytokines, we selected IL-1β and TNF-α for a gene expression analysis using the real-time PCR method in the peripheral blood cells. The obtained results indicate that expression levels for both *IL1B* and *TNFA* genes were significantly higher (*p* = 0.0026 and *p* = 0.025, respectively) in ACS patients compared to the control group. *IL1B* gene expression level was near 2.5-times higher in patients with ACS (average value of 2^−∆Ct^ = 2.1 × 10^−3^) compared to control group (average value of 2^−∆Ct^ = 0.09 × 10^−3^) (Figure 3A). The level of gene expression for TNFA was approximately 3-times higher in patients with ACS (average value of 2^−∆Ct^ = 2.1 × 10^−4^) compared to the control group (average value of 2^−∆Ct^ = 0.69 × 10^−4^) (Figure 3B).

### 2.5. The Expression of 92 Immune Response Associated Genes of Selected Pro-Inflammatory Factors

**Figure 4:**
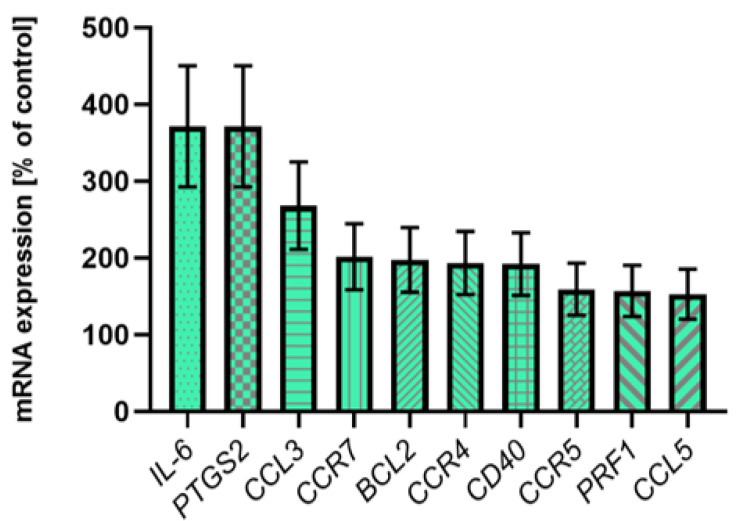
Presentation of the most abundantly expressed genes in screening analysis. Transcripts were distinguished based on their significant expression—over 150% of the control value. All gene expression results were calculated using the 2^−ΔCt^ equation (according to the reference gene encoding 18S rRNA), *n* = 12.

Based on ELISA and real-time PCR results, a screening analysis of the inflammation state expression profile was performed to examine and identify other pro-inflammatory factors that could be associated with ACS to improve the quality of the study. The gene expression for 92 selected pro-inflammatory factors from the peripheral blood cells was measured by the real-time PCR. During screening analysis, it was observed that for 10 genes of pro-inflammatory factors—IL-6, PTGS-2, CCL3, CCR7, BCL-2, CCR4, CD40, CCR5, PRFI, and CCL5—expression was significantly increased (higher than 150% of control) in ACS subjects (Figure 4). In addition, it was noticed that 5 genes—*CCL2*, *CCR2*, *CSF2*, *GZMB*, and *ICOS*—were expressed only in patients with ACS, and all these genes exhibit relatively high expression levels in the compartment from 0.5 × 10^−2^ to 42.0 × 10^−2^ (Table 2). The experimental confirmation of the augmented expression of IL1B and TNFA genes was also presented. The results for the mean gene expression for the remaining genes tested are shown in Table 3.

## 3. Discussion

The multidirectional interactions between leukocytes, blood platelets, and endothelial cells are responsible for the continuous communication between blood cells [32]. The level of platelet–leukocyte aggregates (PLAs) in the bloodstream remains very low under physiological conditions; however, their amount significantly increases in response to various inflammatory states, especially in vascular diseases [33]. In addition, the adhesion of PLAs to the blood vessel wall causes the accumulation of PF-4, which is crucial for the recruitment of monocytes to the damaged endothelium [34], thus promoting the formation of atherosclerotic lesions in vivo [35]. Our study showed that the percentage of formed PLAs (Figure 1A) and the concentration of PF-4 (Figure 2A) in the blood of atherosclerotic patients with non-fatal ACS were significantly increased compared to healthy donors. Furthermore, cytometric measurements of blood platelets showed higher basal expression of P-selectin in the study group in comparison to the control group (Figure 1C), which may indicate their hyperactive state [36]. The possible explanation of overactive platelets may be associated with the presence of atherosclerotic plaques in coronary arteries, which are strongly thrombogenic areas Prolonged stimulation of platelets leads to exfoliation of P-selectin, which can be detected in plasma as soluble (s)P-selectin [37]. Our results showed that enrolled patients had an approximately 15% increased plasma levels of sP-selectin compared to healthy volunteers (Figure 2B). Importantly, sP-selectin retains its functionality in circulation, and the elevated plasma level of sCD62 was confirmed in several inflammatory disorders. It has been reported that sP-selectin promotes leukocyte recruitment at the sites of disrupted endothelium, potentially promoting the early stages of atherogenesis in people at higher risk of vascular disease [38]. What is more, Ridker et al. [39] showed that enhanced sP-selectin concentration is associated with a 25% higher risk of cardiovascular events. 

In addition to P-selectin, blood platelets can form hetero-aggregates with leukocytes by CD40 molecule present on their surface, which plays a critical role in inflammation by stimulating leukocyte and endothelial cells to activate at the site of inflamed vascular, participating in the complex pathway that promotes atherosclerosis. It is reported that the high activity of the CD40/CD40L pathway induces an unstable atherosclerotic plaque phenotype. Our screening analysis showed an increased expression of the *CD40* gene in blood from the ACS patients compared to the controls (Figure 4). 

Blood platelets can interact with all leukocyte subtypes; however, the greatest affinity for the CD62P receptor was shown for monocytes/macrophages and neutrophils [40]. Monocyte subsets and platelet–monocyte aggregates (PMAs) play a critical role in pathological thrombogenesis [41]. Several studies demonstrated the enhanced presence of PMAs in patients with coronary artery disease (CAD), UA, and acute myocardial infarction (AMI) [42]. Our in vitro analysis demonstrated that the level of monocytes in whole blood increased significantly in the study group relative to the control group (Figure 1B). Rivetingly, our screening analysis showed that only patients with ACS expressed CSF2 (Table 2), which is responsible for stimulating stem cells to differentiate into monocytes [43], thus confirming our previously obtained cytometric results. Furthermore, the increased percentage of monocytes in the blood in patients with ACS may be the decisive factor in the enhanced number of PMAs in the bloodstream. The enhanced number of PMAs in whole blood from ACS patients was also confirmed by in vitro studies carried out by Xin et al. [44]. Promotion of PMAs formation was also suggested for monocyte chemoattractant protein 1 (MCP-1), also known as CCL2, which affects monocyte migration and their increased accumulation in the lumen of blood vessels [45]. Furthermore, it was confirmed that the CCL2/CCR2 axis regulates the macrophage accumulation at the site of atherosclerotic changes [46]. Results from our study showed that expression of *CCR2* and *CCL2* on the mRNA level was shown only in atherosclerotic patients with non-fatal ACS (Table 2), thus confirming the association of PMAs formation with atherosclerosis and ACS. Promotion of late-stage atherosclerosis was also shown for another chemokine, CCR5 [47], the expression of which was higher in the study group compared to the control group (Figure 4). 

According to available literature, several chemokines in atherosclerotic lesions have been reported, including CCL3/MIP-1a (Macrophage Inflammatory Proteins 1a) and CCL5/RANTES (Regulated upon Activation, Normal T-cell Expressed, and Secreted). These chemokines may recruit monocytes, memory T cells, and dendritic cells to the inflammation sites and are associated with the risk of short-term mortality in ACS patients. Multiplex analysis of the baseline serum level of chemokine proteins conducted by de Jager et al. showed that a significantly enhanced level of CCL3 and CCL5 increases the mortality risk in patients with ACS [48]. Our screening studies in whole blood in ACS patients confirmed increased mRNA expression for CCL3, CCL5 and chemokine receptors CCR4, CCR5, and CCR7 (Figure 4).

Based on the review of the available literature, we selected the most critical pro-inflammatory cytokines for evaluation. Results showed the augmented concentration of IL-1β, TNF-α, IL-2, and INF-γ in the plasma of the study group compared to healthy donors. No significant differences were observed for TGF-β and INF-α (Table 1). The increased level of IL-1β in human plasma patients with atherosclerosis was shown in An et al. study [49]. Furthermore, the CANTOS clinical trial showed that inhibition of IL-1β results in a reduced rate of cardiovascular events [27]. TNF-α is another major pro-inflammatory cytokine that enhances the risk of atherosclerotic thickening causing triglyceride and glucose metabolism disorders and elevating the risk of recurrent ACS disease [50,51]. Maury et al. showed that the concentration of TNF-α was correlated with the severity of the myocardial infarction (MI) patients and infarct volume [52]. Real-time PCR analysis performed in our study showed an increased in IL1B (Figure 3A) and TNFA (Figure 3B). Furthermore, results obtained from IL-2 protein analysis showed significant elevation among ACS patients compared to healthy donors. Similar results were shown in the Mazzone et al. study [53].

Interestingly, results obtained on the mice model injected with IL-2 showed a significant increase in the average atherosclerotic lesion size [54]. Our results from performed screening analysis showed overexpression of IL-2 at the mRNA level in ACS. Our proteomic analysis also showed that patients with ACS had an augmented concentration of IFN-γ, which is a well-known cytokine highly expressed in atherosclerotic lesions. Studies showed that IFN-γ could induce the generation of reactive oxygen species (ROS) that induce oxidative modification of low-density lipoprotein (LDL), which is crucial for the formation of atherosclerotic plaque [55]. Similar results were also shown by Ranjbaran et al. in a study in which IFN-γ was elevated in plasma patients with coronary atherosclerosis [56]. Surprisingly, our proteomic analysis did not show any statistically significant differences in the concentration of TGF-β, which is an essential anti-inflammatory cytokine that controls proliferation and differentiation in most cell types and is one of the main drivers of fibrotic changes and endothelial–mesenchymal transition [57]. The possible explanation may be associated with the early blood sampling from ACS patients.

Results from our screening analysis showed an increased expression of *PRF1* in the study group (Figure 4), whereas *GZMB* expression was present only in the study group (Table 2). Hiebert et al. proved crucial roles for granzyme B/perforin GZMB/PRF cytotoxic pathway in the atherosclerosis pathogenesis that goes above the classical apoptotic pathway with additional implications in plaque development. They showed that high levels of GZMB and PRF in the aorta are associated with atherosclerotic plaque instability in vivo [58]. Thus, higher *GZMB* and *PRF1* gene expression in ACS patients may increase levels of these proteins in the blood and potentially lead to the rupture of atherosclerotic plaques in the coronary vessels. The contribution of PRF and GZMB molecules in the context of ACS pathogenesis is not fully explained; however, our results may highlight a hypothetic direction for the future studies focused on the cytotoxic pathway analysis and provide a new insight linked with a better understanding of the plaque rapture physiology.

Performed screening analysis demonstrates a high expression of the *PTGS2* gene in the study group (Figure 4), known as a cyclooxygenase-2 (COX-2) [59]. It has been shown that increased COX-2 levels in macrophages may dictate a predominant pathway of arachidonate metabolism leading to increased biosynthesis of prostaglandin E2 (PGE2) and PGE2-dependent matrix metalloproteinases (MMPs), which has the potential to cause acute carotid plaque disruption [60]. Interestingly, our results also showed that only patients with ACS presented the expression of an inducible co-stimulatory (ICOS), which is considered as a protective rather than a potent pro-atherosclerotic factor [61]. This phenomenon may lead to the assumption that patients with ACS already had a reasonably advanced stage of atherosclerosis, thus suggesting that protective genetic processes in the human body have already been triggered to reduce further progression of atherosclerosis. 

We are fully aware of the crucial limitations of this study. One of them is the limited number of patients involved in this study group. However, patients qualifying for our study were limited by many risk factors and by the time between the presence of thrombotic event and blood collection, which was supposed to provide the least possible effect of the applied pharmacotherapy. In addition, the direct cellular origin of the pro-inflammatory factors remains unclear, and the detailed analysis of all leukocyte subsets, platelets, and endothelial cells requires further analysis in the larger group. Analysis was performed at a single time point, which may lead to the omission of the changes caused by regulation of gene expression which lasts further during the following phases of the disease. Some of the altered expressed inflammatory molecules are poorly described in the context of ACS pathogenesis (i.e., GZMB, PRF, and ICOS), while their involvement in vascular disease has been confirmed. Results obtained from our preliminary study may constitute an interesting concept for further, detailed research on the molecular pathways possibly involved in the pathogenesis of atherosclerosis and ACS.

## 4. Materials and Methods

### 4.1. Patients

Blood samples were collected from 12 patients (aged 57 ± 11) with coronary angiography diagnosed with atherosclerosis and ACS admitted to Intensive Cardiac Therapy Clinic, Department of Interventional Cardiology and Electrocardiology, Medical University of Lodz, Poland. The blood was collected in CPDA-1 (citrate-phosphate-dextrose-adenine 1) Sarstedt^®^ tubes (Nümbrecht, Germany) within 3 h from admission to the hospital. Patients with confirmed ACS enrolled in the study did not have typical cardiovascular disease risk factors, such as high cholesterol, lipid anomalies, diabetes mellitus (IFG and IGT), metabolism disorders of purines, high level of fibrinogen, immune disorders (IgM and IgG levels), extreme obesity (BMI >35), hypertension, renal failure, hypothyroidism, connective tissue disease, cancer at the time of the interview, consumption of narcotics (drugs, boosters), or alcohol. Furthermore, patients were not previously diagnosed with any cardiovascular diseases. The optical coherence tomography was not performed. During hospitalization, patients were treated with anti-platelet drugs (Aspirin, Ticagrelor or Clopidogrel, and Epitifibatide if indicated), statins (Rosuvastatin), anti-coagulants (Heparin, Enoxaparin), and supportive medicaments (Morphin, Nitroglycerin, Metoprolol, and Furosemide). During the coronary angiography, patients received Ultravist as the contrast medium. The possible interactions caused by administrated medicaments were taken into consideration during the experiment design. 

The control group was homologous to the study group in terms of number (*n* = 12), gender, and age. Blood samples from donors were collected by qualified medical staff in the Center of Laboratory Diagnostics in Lodz, Poland. Before inclusion, all volunteers classified as appropriate for the study were selected based on medical history and were subjected to the same medical tests as a study group of volunteers who had never been diagnosed with ACS nor suffered from any chronic inflammatory disease. The healthy donors did not take any medicines for a minimum of 2 weeks before blood collection and enrollment in the study. 

The study was conducted according to the guidelines of the Declaration of Helsinki and approved by the Bioethics Committee of the University of Lodz with Resolution No. 14/KBBN-UŁ/II/2016 on November 10, 2016.

All information on the population characteristics and exclusion criteria are included in Table 4. In addition, all participants and/or their legal guardians were informed about their rights, and their consents were obtained. 

### 4.2. Analysis Using Flow Cytometry

At the first analysis stage, the erythrocytes in fresh blood samples were lysed in BD FACS Lysing Solution (Becton Dickinson, San Diego, CA, USA). After 1 h lysis, the appropriate samples were stained with specific murine monoclonal IgG and incubated for 30 min in the dark at 25 °C. Subsequently, 500 µL of CellFix (1%) solution (Becton Dickinson, San Diego, CA, USA) was added to fix the samples, and each sample was centrifuged (5000× *g*, for 10 min.). Before the analysis, the obtained sediment was dissolved in 500 µL of NaCl (0.9%). For platelet–leukocyte aggregates (PLAs) analysis, two different types of specific antibodies—antibody against platelet CD61 conjugated with fluorescein isothiocyanate (FITC) (Catalog number: 347407; Becton Dickinson, San Diego, CA, USA) and an antibody against CD45 (leukocyte marker) conjugated with phycoerythrin (PE) (555483; Becton Dickinson, San Diego, CA, USA)—were used. The surface expression of P-selectin (CD62P) was determined using a PE-conjugated antibody against–CD62P (555524; Becton Dickinson, San Diego, CA, USA). All antibodies for flow cytometry analysis were purchased from Becton Dickinson (San Diego, CA, USA). Blood platelets were gated based on bright CD61 fluorescence (FL1), and CD61+ objects with a fluorescence parameter on a logarithmic scale above 10^1^ were considered platelets. All subtypes of leukocytes were gated based on bright CD45 fluorescence (FL2), and objects CD45+, which had a fluorescence parameter above 10^1.5^, were considered leukocytes. The fluorescence of 15,000 CD61+ was measured each time. The percentage of CD61+/CD45+ (PLAs) was determined comparative to the whole number of CD45+ subjects, and the percentage of CD61+/CD62P+ (blood platelet with P-selectin expression) were calculated comparative to the whole platelets (CD61+) population present in each sample. An isotype-matched IgG-control determined nonspecific membrane immunofluorescence and background fluorescence. The analysis of PLAs, monocyte participation, and examination of platelet surface expression of P-selectin in non-activated whole blood samples was performed using CUBE 6 flow cytometry with CyView Software v.1.5.5.8 (Partec, Görlitz, Germany).

### 4.3. Cytokine Level Analysis

The fresh whole blood samples with CPDA-1 anticoagulant collected by Sarstedt^®^ tubes (Nümbrecht, Germany) were centrifuged (3305× *g*, for 12 min at room temperature) to obtain platelet-poor plasma (PPP). The following cytokine levels—IL-1β (Product code: 3416-1H-6), IL-2 (3445-1H-6), IFN-γ (3420-1H-6), IFN-α (3425-1H-6), TGF-β (3550-1H-6), and TNF-α (3510-1H-6)—were measured using commercial Human ELISA Development Kit (Mabtech, Nacka Strand, Sweden) following all instructions provided in the manufacturer′s recommendations. The measurements were based on 3,3′,5,5′-Tetramethylbenzidine (TMB) substrate oxidation and were executed using MaxiSorp Plates (Nunc, Roskilde, Denmark). The absorbance was measured at a wavelength of 450 nm using the SPECTROstar Nano Microplate Reader (BMG Labtech, Ortenberg, Germany).

### 4.4. PF-4 Level Analysis

The level of PF-4 was measured in PPP using a commercial Human PF-4 ELISA Kit (Code: ELH-PF4-1; RayBiotech, Norcross, GA, USA) following all instructions provided in the manufacturer′s protocol. The level of PF-4 in plasma was determined from a standard curve expressed as pg/mL. The absorbance was measured at a wavelength of 450 nm using the SPECTROstar Nano Microplate Reader (BMG Labtech, Ortenberg, Germany).

### 4.5. Soluble P-Selectin Measurement

The level of soluble P-selectin (sP-selectin) in human plasma was measured using P-selectin (soluble) Human ELISA Kit (Catalog number: BMS219-4; Invitrogen, Waltham, MA, USA) according to the manufacturer′s protocol. The level of sP-selectin in plasma was determined from a standard curve expressed as ng/mL. The absorbance was measured at a wavelength of 450 nm using the SPECTROstar Nano Microplate Reader (BMG Labtech, Ortenberg, Germany).

### 4.6. Isolation of Total RNA and Reverse Transcription

Total RNA was isolated from whole frozen blood samples (−80 °C) using TRI Reagent^®^ (Sigma-Aldrich, Saint Louis, MO, USA). Purification of the RNA-containing aqueous phase was then conducted using an InviTrap^®^ Spin Universal RNA Mini Kit (Stratec Molecular GmbH, Berlin, Germany). To estimate the quantity and purity of the obtained RNA, the Synergy HTX Multi-Mode Microplate Reader, equipped with a Take3 Micro-Volume Plate (BioTek Instruments, Inc. Winooski, VT, USA), was used. To obtain the cDNA, the reverse transcription of the total RNA the iScript^TM^ cDNA Synthesis Kit (Bio-Rad, Hercules, CA, USA) was used. All steps were performed following the manufacturers’ recommendations.

### 4.7. Analysis of Gene Expression

For the analysis of gene expression for IL-1β and TNF-α molecules, the following TaqMan probes were used: Hs01555410_m1 for the human *IL1B* gene, Hs00174128_m1 for the human *TNFA* gene, and Hs99999901_s1 for the human *18S rRNA* used as an endogenous control) (Thermo Fisher Scientific, Waltham, MA, USA). Screening analysis of the expression profile of 92 genes associated with the function of the immune system was measured using the commercial test kit TaqMan^®^ Array 96-Well Plate Human Immune Response (Thermo Fisher Scientific, Waltham, MA, USA). Although the provided microplate contains 96 wells, 4 wells are reserved for reference genes (*18S rRNA, GAPDH, HPRT1, GUSB*). However, we used only 18S rRNA as an internal control, so that results can be compared with those obtained using TaqMan probes in this and our previously published papers. Gene expression measurements were made on the Real-Time PCR–The CFX96™ Touch System (Bio-Rad, Hercules, CA, USA) using a TaqMan Universal Master Mix II, no UNG (Thermo Fisher Scientific, Waltham, MA, USA). The cDNA was obtained from the mRNA isolated from the whole blood of patients with ACS and the control group was used for the analysis. All procedures were executed following the manufacturer’s protocols. To calculate the relative expressions of the genes studied, the equation 2^−ΔCt^, where ΔCt = Ct_target gene_ − Ct_18S rRNA_, was used.

### 4.8. Statistical Analysis

All the experiments carried out were duplicated. The statistical significance between samples and controls was determined using the Mann–Whitney U test. The *p* < 0.05 values are considered statistically significant. A statistical power analysis was performed using the following assumptions: variability within the group based on flow cytometry analysis—20% [32,62]; the dimension of the experimental effect—20% [63] (this change is considered as interesting in clinical/diagnostic practice). This statistical power was established at 70%. Distribution normality was examined using the Shapiro–Wilk test, and then, the significance of the difference between studied values was determined based on the Mann–Whitney test or Student’s *t*-test. For normally distributed data, the results were presented as the mean ± SD, while the non-normally distributed results were presented as the median with an interquartile range. All data were analyzed using the StatsDirect^®^ statistical software Version 2.7.2 (StatsDirect software, Cheshire, UK), and all figures were prepared in the GraphPad Prism 7.0 (GraphPad Software, Inc., San Diego, CA, USA).

## 5. Conclusions

In summary, the results of our research demonstrates altered gene expression of novel inflammatory factors, suggests their potential role in the progression of atherosclerosis, and underlines the necessity of further, more detailed genetic analysis. Furthermore, the cellular interaction mentioned in our manuscript may constitute an essential target for future pharmacotherapies. Correct phenotypic assessment of the microvascular environment, which would identify the complexity of cellular interactions and determine the genetic variability of atherosclerosis, could contribute to better clinical evaluation of ACS patients in the future and allow to diagnose a pathological process in the human body, thus reducing the mortality and morbidity of cardiovascular diseases.

## Figures and Tables

**Table 1:** The level of pro-inflammatory cytokines IL-1β, IL-2, TNF-α, IFN-α, IFN-γ, and TGF-β.

Cytokine	Control Group	ACS	*p*-Value	Increment
IL-1β (pg/mL)	21.7 (±12.25)	45.50 (±15.39)	***p* = 0.0003**	↑ 216%
TNF-α (pg/mL)	356.33 (±105.53)	752.33 (±169.20)	***p* < 0.0001**	↑ 211%
IL-2 (pg/mL)	95 (89–121.30)	126 (111.5–156)	***p* = 0.011**	↑ 133%
IFN-γ (pg/mL)	206.50 (204.50–299.80)	304 (245–340)	***p* = 0.033**	↑ 128%
TGF-β (pM)	100 (86.25–98.75)	97 (94.25–102)	*p* = 0.678	N/S
IFN-α (pg/mL)	11.50 (9.25–19.75)	16 (12.75–24.75)	*p* = 0.091	N/S

Pro-inflammatory cytokines concentration was measured in plasma from ACS patients (*n* = 12) and healthy subjects (*n* = 12). For normally distributed data, the Student’s *t*-test was used and the results were presented as the mean ± SD, while for the non-normally distributed results, the Mann–Whitney U test was used, and data were presented as the median with interquartile range. Statistically significant *p*-values (*p* < 0.05) are marked in bold, while N/S means no significance.

**Table 2:** The mRNA expression of pro-inflammatory molecules exclusively presented in ACS patients.

No.	Assay ID	Gene Symbol	Gene Expression (2^−ΔCt^) ± SD in ACS
1	Hs00234140_m1	*CCL2*	0.5 ± 0.3 [× 10^−2^]
2	Hs00174150_m1	*CCR2*	2.0 ± 1.3 [× 10^−2^]
3	Hs00171266_m1	*CSF2*	1.0 ± 0.6 [× 10^−2^]
4	Hs00188051_m1	*GZMB*	42 ± 27 [× 10^−2^]
5	Hs00359999_m1	*ICOS*	1.5 ± 1 [× 10^−2^]

The results are represented as a mean of 2^−ΔCt^ (according to the reference gene encoding 18S rRNA) ± SD, *n* = 12.

**Table 3:** Screening analysis of the expression of the remaining genes associated with the function of the immune system. All results are expressed as a mean of 2^−^**^Δ^**^Ct^ (according to the reference gene encoding 18S rRNA); N/A–not available. The values of each mean gene’s expression in the study group are marked in red, while in the control group in blue.

No.	Assay ID	Gene Symbol	Gene Expression (2^−ΔCt^) for Control	Gene Expression (2^−ΔCt^) for ACS
1	Hs00174092_m1	*IL1A*	N/A	N/A
2	Hs00174097_m1	*IL1B*	2.304	2.021
3	Hs00174114_m1	*IL2*	0.011	N/A
4	Hs00174117_m1	*IL3*	N/A	N/A
5	Hs00174122_m1	*IL4*	N/A	N/A
6	Hs00174200_m1	*IL5*	N/A	N/A
7	Hs00174202_m1	*IL7*	N/A	N/A
8	Hs00174103_m1	*IL8*	3.148	3.398
9	Hs00174125_m1	*IL9*	N/A	N/A
10	Hs00174086_m1	*IL10*	N/A	N/A
11	Hs00168405_m1	*IL12A*	0.026	0.015
12	Hs00233688_m1	*IL12B*	N/A	N/A
13	Hs00174379_m1	*IL13*	N/A	N/A
14	Hs00174106_m1	*IL15*	N/A	N/A
15	Hs00174383_m1	*IL17A*	N/A	N/A
16	Hs00155517_m1	*IL18*	0.034	0.029
17	Hs00171149_m1	*CCL19*	N/A	N/A
18	Hs00171041_m1	*CXCR3*	N/A	N/A
19	Hs00171042_m1	*CXCL10*	0.025	0.015
20	Hs00171138_m1	*CXCL11*	0.016	N/A
21	Hs00174164_m1	*CSF1*	0.007	0.005
22	Hs00357085_g1	*CSF3*	N/A	N/A
23	Hs00234174_m1	*STAT3*	0.630	0.362
24	Hs00174517_m1	*NFKB2*	0.315	0.324
25	Hs00233284_m1	*IKBKB*	0.065	0.035
26	Hs00167894_m1	*CD3E*	1.046	1.380
27	Hs00181217_m1	*CD4*	0.239	0.271
28	Hs00233520_m1	*CD8A*	0.509	0.530
29	Hs00174333_m1	*CD19*	0.057	0.014
30	Hs00166229_m1	*IL2RA*	0.085	0.021
31	Hs00174796_m1	*CD28*	0.130	0.115
32	Hs00233552_m1	*CD38*	0.074	0.031
33	Hs00365634_g1	*PTPRC*	3.397	2.818
34	Hs00154355_m1	*CD68*	1.370	0.653
35	Hs00175478_m1	*CD80*	0.009	0.007
36	Hs00199349_m1	*CD86*	0.443	0.347
37	Hs00175480_m1	*CTLA4*	0.063	0.070
38	Hs00163934_m1	*CD40LG*	0.133	0.017
39	Hs00219575_m1	*HLA-DRA*	7.697	9.746
40	Hs99999917_m1	*HLA-DRB1*	N/A	N/A
41	Hs00203436_m1	*TBX21*	0.057	0.049
42	Hs00188346_m1	*TNFRSF18*	N/A	N/A
43	Hs00167248_m1	*NOS2*	N/A	N/A
44	Hs00169141_m1	*BCL2L1*	0.431	0.239
45	Hs00180269_m1	*BAX*	0.410	0.244
46	Hs00164932_m1	*ICAM1*	0.111	0.135
47	Hs00174583_m1	*SELP*	0.218	0.110
48	Hs00174057_m1	*SELE*	N/A	N/A
49	Hs00157965_m1	*HMOX1*	0.194	0.055
50	Hs00189742_m1	*LRP2*	N/A	N/A
51	Hs00167927_m1	*CYP1A2*	N/A	N/A
52	Hs00167982_m1	*CYP7A1*	N/A	N/A
53	Hs00174143_m1	*IFNG*	0.008	0.010
54	Hs00246266_m1	*GNLY*	4.939	7.386
55	Hs00163653_m1	*FAS*	0.242	0.296
56	Hs00181225_m1	*FASLG*	N/A	N/A
57	Hs00171257_m1	*TGFB1*	2.304	2.403
58	Hs00232222_m1	*SMAD3*	0.035	0.037
59	Hs00178696_m1	*SMAD7*	0.075	0.035
60	Hs00365052_m1	*FN1*	N/A	N/A
61	Hs00163811_m1	*C3*	0.014	0.006
62	Hs00174128_m1	*TNF*	0.062	0.074
63	Hs00236874_m1	*LTA*	N/A	N/A
64	Hs00174179_m1	*ACE*	N/A	N/A
65	Hs00173626_m1	*VEGFA*	N/A	N/A
66	Hs00161707_m1	*SKI*	0.035	0.047
67	Hs00156373_m1	*CD34*	N/A	N/A
68	Hs00241341_m1	*AGTR1*	N/A	N/A
69	Hs00169126_m1	*AGTR2*	N/A	N/A
70	Hs00174961_m1	*EDN1*	0.043	N/A
71	Hs00171455_m1	*LIF*	N/A	N/A
72	Hs00209771_m1	*LY96*	0.043	0.618
73	Hs00236988_g1	*MIF*	6.889	5.080
74	Hs00190046_m1	*NFATC3*	1.664	1.735
75	Hs00190037_m1	*NFATC4*	N/A	N/A
76	Hs00236998_m1	*PF4*	18.692	14.772
77	Hs00374292_m1	*SYK*	N/A	N/A

For genes with N/A results we did not observe any product formation, thus the fluorescent level (RFU) did not exceed the threshold which was automatically calculated by the thermocycler during analysis.

**Table 4:** Clinical characteristics of the ACS patients and control group.

Characteristic [Clinical Standards]	Control Group (*n* = 12)	Study Group (ACS) (*n* = 12)
Age	55 ± 6.61	57 ± 11 (*p* = 0.100; n.s.)
Gender (M/F)	M8/F4	M8/F4
BMI [<30]	26.40 ± 6.61	21.9 ± 4.85 (*p* = 0.413; n.s.)
Triglycerides [<2.5 mmol/L]	1.18 ± 0.54	2.17 ± 0.82 (***p* = 0.005**)
Total cholesterol [3–5 mmol/L]	4.02 ± 1.34	4.68 ± 1.12 (*p* = 0.196; n.s.)
HDL [>1 mmol/L]	1.31 ± 0.53	1.17 ± 0.21 (*p* = 0.700; n.s.)
LDL [<2.9 mmol/L]	2.89 ± 1.09	1.54 ± 0.97 (*p* = 0.413; n.s.)
Leukocytes on admission [4–11 × 10^3^/μL]	7.78 ± 2.52	9.63 ± 2.42 (***p* = 0.027**)
Erythrocytes on admission [4–6 × 10^6^/μL]	5.08 ± 0.41	4.72 ± 0.66 (*p* = 0.118)
Platelets on admission [150–400 × 10^3^/μL]	316.17 ± 119.70	242.3 ± 87.2 (*p* = 0.098; n.s.)
Creatinine [64–104 μmol/L]	83.94 ± 15.70	84.25 ± 14.01 (*p* = 0.960; n.s.)
Troponin peak (hs-TnT) [<14 ng/L]	13.34 ± 0.23	2778 ± 2361.33 (***p* = 0.0005**)
ALT [0–45 UI]	23.92 ± 12.80	30.07 ± 13.18 (*p* = 0.259)
AST [0–35 UI]	21.30 ± 7.12	33.44 ± 15.07 (***p* = 0.019**)
Glucose [4.1–5.5 mmol/L]	5.07 ± 0.60	5.40 ± 0.44 (*p* = 0.131)
GFR [ml/min./1.73 m^2^]	94 ± 14	93 ± 9 (*p* = 0.735)
NSTEMI/STEMI	-	4/8
Localization of culprit lesion	-	Cx (*n* = 5); RCA (*n* = 4); LCA (*n* = 1); LAD (*n* = 2)
Statins administration	-	Patients before admission to hospital were not treated with statins. All patients received first dose of statin (at least 20 mg dose of Rosuvastatin) during the first 24 h of ACS.

Abbreviations: ALT–alanine transaminase; AST–aspartate transaminase; BMI–body mass index; Cx–circumflex artery; F–female; GFR–glomerular filtration rate; HDL–high-density lipoprotein; hs-TnT–high-sensitivity troponin T; LAD–left ante-rior descending artery; LCA–left main coronary artery; LDL–low-density lipopro-tein; M–male; NSTEMI–non-ST-elevation myocardial infarction; RCA–right coronary artery; STEMI–ST-elevation myocardial infarction. Statistically significant *p*-values (*p* < 0.05) are marked in bold.

## Data Availability

All data generated or analyzed during this study are included in this published article.

## Abbreviations

5-HT–5-hydroxytryptamine; ACS–acute coronary syndrome; ALT–alanine transaminase; AMI–acute myocardial infarction; AST–aspartate transaminase; BMI–body mass index; CAD–coronary artery disease; COX-2–cyclooxygenase-2; CPDA-1–citrate-phosphate-dextrose-adenine 1; Cx–circumflex artery; EGF–epidermal growth factor; F–female; GFR–glomerular filtration rate; GZMB–granzyme B; HDL–high-density lipoprotein; hs-TnT–high-sensitivity troponin T; ICOS–inducible co-stimulatory; IFN-α–interferon α; IFN-γ–interferon γ; IL-β–interleukin 1β; LAD–left ante-rior descending artery; LCA–left main coronary artery; LDL–low-density lipopro-tein; MCP-1–monocyte chemoattractant protein 1; MIP-1a–macrophage Inflammatory Proteins 1a;M–male; MMPs–matrix metalloproteinases; NSTEMI–non-ST-elevation myocardial infarction; PF-4–platelet factor 4; PGE2–prostaglandin E2; PLAs–platelet–leukocyte aggregates; PMAs–platelet–monocyte aggregates; PRF–perforin; RANTES–regulated upon activation, normal T-cell expressed, and secreted; RCA–right coronary artery; ROS–reactive oxygen species; sP-selectin–soluble P-Selectin; STEMI–ST-elevation myocardial infarction; TGF- –transforming growth factor β; TGF-β–tumor necrosis factor β; TNF-α–tumor necrosis factor α; UA–unstable angina; WHO–World Health Organization.
